# The global potential for carbon capture and storage from forestry

**DOI:** 10.1186/s13021-016-0044-y

**Published:** 2016-02-26

**Authors:** Yuanming Ni, Gunnar S. Eskeland, Jarl Giske, Jan-Petter Hansen

**Affiliations:** 1 Department of Business and Management Science, Norwegian School of Economics, Helleveien 30, 5045 Bergen, Norway; 2 Department of Biology, University of Bergen, 5020 Bergen, Norway; 3 Department of Physics and Technology, University of Bergen, Allegt. 55, 5007 Bergen, Norway; 4 Centre for Applied Research (SNF), Helleveien 30, 5045 Bergen, Norway

**Keywords:** Carbon capture and storage, Simulations, Global forest

## Abstract

**Background:**

Discussions about limiting anthropogenic emissions of CO$_2$ often focus on transition to renewable energy sources and on carbon capture and storage (CCS) of CO$_2$. The potential contributions from forests, forest products and other low-tech strategies are less frequently discussed. Here we develop a new simulation model to assess the global carbon content in forests and apply the model to study active annual carbon harvest 100 years into the future.

**Results:**

The numerical experiments show that under a hypothetical scenario of globally sustainable forestry the world’s forests could provide a large carbon sink, about one gigatonne per year, due to enhancement of carbon stock in tree biomass. In addition, a large amount of wood, 11.5 GT of carbon per year, could be extracted for reducing CO$_2$ emissions by substitution of wood for fossil fuels.

**Conclusion:**

The results of this study indicate that carbon harvest from forests and carbon storage in living forests have a significant potential for CCS on a global scale.

## Background

According to the intergovernmental panel on climate change (IPCC), a reduction of the anthropogenic emissions of CO$_2$ to the atmosphere is necessary to avoid global warming beyond two degrees [1]. When also considering the projected population and consumption growth [2], the CO$_2$ reductions needed are daunting. It will require a transition to CO$_2$ free energy sources in many applications, and/or CCS from facilities such as fossil-based power plants. CO$_2$ free energy requires a significant build-up of nuclear and/or renewable power production, which involves large initial economic investments [3]. For CCS, a range of alternatives exist, each with its particular challenges. Industrial CCS requires energy and costly facilities and the captured gas has to be transported and stored in stable geological formations [4]. The cost of the capture process itself is estimated to be in the range of 40–70 euros/tonne CO$_2$, depending on technology [5].

The scale of the problem should not be underestimated: to reach the less than two degree goal of IPCC, the annual CO$_2$ emissions must be reduced from the current level of 10 GT of carbon per year (GtC/year) to 5–6 GtC/year by 2050. Hence, the new energy sources need to deliver up to 5 terrawatts (TW) or above [5]. To contribute at this scale, the sequestrated amount of carbon by industrial CCS has to be of order 1 GtC/year or more, while the total amount of carbon stored so far is only a few tens of megatonnes, ie., a few per mille of the necessary amount. It is therefore important to consider alternative options. One alternative is to use the photosynthesis to increase the carbon sink by increased magnitude of the world’s forests. Another option is to increase the carbon uptake by letting forest stands grow for longer periods [6]. Two other alternatives could be a large scale deployment of artificial photosynthesis [7], or to increase the carbon uptake of the oceans by adding active absorbers. However, the latter alternatives may involve high risk for unexpected drawbacks [8].

The annual sink of the world’s forests has been estimated to be about 2.4 GtC [9]. Taking advantage of photosynthesis in forests requires global schemes for reducing deforestation in combination with planting and replanting programs. Also, a possibility is modified harvest and management. Recently, it was argued that this large-scale planting action may also have a negative total climate effect since the greening of open land areas will reduce the albedo of the earth [10]. An alternative would be to collect wood material at a constant rate and then store it. Independent strategies based on this idea were suggested a few years ago [11, 13]. In this context the storage problem would be small. Dry wood contains about 50 % carbon [14] and can be stored for example in decommissioned coal mines or in facilities near the forests in the long term. By using timber in fairly long-lasting applications (buildings, furniture), carbon storage could be even less expensive and more attractive. The global potential of this option was initially estimated to be as much as 5–15 GtC/year [11] and later estimated to be around 1–3 GtC/year [12] when land use, protection consideration and other factors were taken into account. This estimate is based on simulations using a model based on global carbon fluxes.

Storing carbon as standing forests or from harvested wood has long been recognized as a CCS option: for example, Schroder et al. estimated that 15–36 GtC could be stored in tropical plantations and 50–100 GtC sequestrated on a global scale [15, 16]. A detailed analysis of the Eastern US woodlands shows that 176 megatonnes of wood may be harvested annually without diverting current wood products, damaging habitat, or reducing terrestrial carbon sink [17]. A calculation by Lehmann [18] indicates that an equivalent of about 10 % of the US fossil fuel emissions may be harvested from biomass and stored as biochar. Based on simulating the growth of uneven-aged mixed beech-spruce stands in the temperate region, Kraxner et al. [19] found that 1–2 tonnes/year/ha can on average be extracted for storage. Several model studies also include economic aspects of forest management and carbon storage [20–22]. In terms of policies, our analysis goes in the same direction as for instance Hoel et al. [23]. They argue that forests and forest products may serve the climate better if valued not only as renewables but also for carbon storage purposes. While the literature is vast regarding regional and tree-specific studies, see e.g., [19, 24], to the best of our knowledge there has been no attempt to address the issue dynamically based on the nonlinear growth of trees to obtain numerical results with harvesting schemes for global forests as a whole. For example, by scaling up the simulation of Kraxner et al. [19], one attains an estimate of 0.7–1.4 GtC per year as the CCS potential of the temperate forests. However, upscaling from detailed growth models needs to be performed with caution.

Here, we attempt to address this problem by modeling global potential for carbon storage in biomass based on a plant-harvest forestry program. It is assumed that a fraction of the forest is harvested and replanted every year. As a starting point we assume that forest growth in the boreal, temperate and tropical regions is latitude-dependent. Reasonable assumptions are also made regarding future reforestation and deforestation for different zones. The results are then directly based on non-linear forest growth and the instantaneous status of the world forests.

## Results and discussion

We here demonstrate four applications of our model (described in the "Methods" section) in terms of strategies for harvesting, reforestation (replanting) and afforestation (new planting). The present forest area for each region is discretized to a sufficiently large number of initial forest areas. For example, when taking a discretized area size of 105 hectares, the simulation starts with 21111, 7394 and 11825 areas representing global forests in tropical, temperate and boreal zones respectively [25]. The carbon content in living biomass per ha is used [9] to reach an estimated implied initial average age of 94, 57 and 65 years for tropical, temperate and boreal zone trees.

**Fig. 1:**
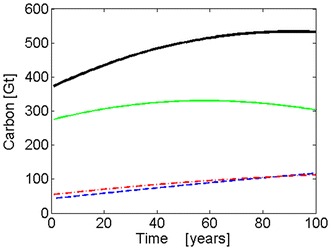
Dynamic carbon content in tropical zone (*green full*), temperate zone (*blue dashed*), boreal zone (*red dashed-dot*) and world forest (*black thick full*) forecast with our model. Parameters: deforestation rate of 0.44 % in the tropical zone, current expansion rates of 0.1 % in the boreal zone and 0.264 % in the temperate zone [25]. The initial year is 2010

In Fig. 1, we display the carbon content of world forests 100 years into the future, assuming constant (present) rates of deforestation and expansion without additional harvesting. Starting from 372 GtC, total carbon content is seen to increase to 530 GtC. This is a net increase of 158 GtC in spite of a present deforestation rate of 0.44 % per year in the tropical region [25]. The increased carbon content comes from afforestation in the temperate and boreal zones, combined with increased carbon storage due to growth in maturing tropical trees. The assumption of constant growth rates for 100 years into the future may clearly be questioned. In the extreme case of a complete stop in tropical deforestation from today, the forest carbon amount would for example increase by up to 230 GtC within 100 years.

With non-harvest (status quo), as a background, we are now ready to model forest development under four alternative harvest-replanting scenarios. All harvesting strategies assume immediate replanting so that the land remains functioning as forest land. We also assume that tropical deforestation is alleviated from 0.44–0.3 % during the whole simulation period. The first two simulations assume harvesting parameters of 0.3 and 0.45 %, respectively, for all three zones. In the third strategy, we reduce the harvesting parameter to 0 in the tropical zone, and increase it to 0.8 % in the two other zones. The reduced harvesting rate in the tropics is motivated by the special soil feature in tropical areas. Due to relative high temperature all year round, the decomposition of forest residues happens fast, and this results in a rather thin layer of soil. Harvesting and storing wood away under these conditions may cause significant reduction in ground soil, motivating the non-harvest strategy. In the final simulation an extra plantation program is introduced so that every year a specified fraction of new forest area is added into the model. This is to explore the potential under a large-scale policy change of using forests as a CCS method.

**Fig. 2:**
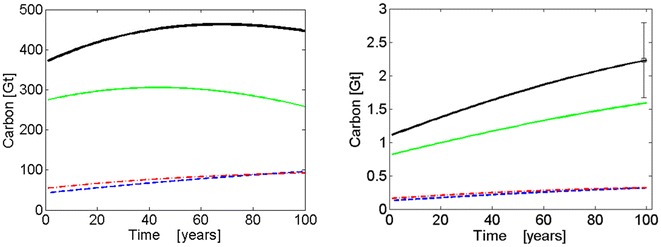
*Left panel* Total forest carbon development in tropical zone (*green full*), temperate zone (*blue dashed*), boreal zone (*red dashed-dot*) and world forest (*black thick full*). *Right panel* Yearly harvested carbon development in tropical zone (*green full*), temperate zone (*blue dashed*), boreal zone (*red dashed-dot*) and world forest (*black thick full*) with indicative error bar (*square*) at year 100. Parameters: harvesting rate of 0.3 % in all zones; deforestation rate of 0.3 % in tropical zone; current expansion rates of 0.1 % in the boreal and 0.264 % in the temperate zones

In simulation 1 with 0.3 % harvest rate the total forest carbon increases from 372 to 447 GtC over 100 years (Fig. 2). Considering the harvested fraction of 0.3 %, it seems that these 1.5–2 GtC can be extracted every year without harming forest production. The total carbon sequestration can thus be 2.25–2.75 GtC/year from both standing stock and harvested wood. The major contribution comes from the tropical zone, due to its stock size and productivity. At the end of the period, the constant deforestation rate finally brings down the total carbon stock in the tropical zone, while the other two zones still display growth.

**Fig. 3:**
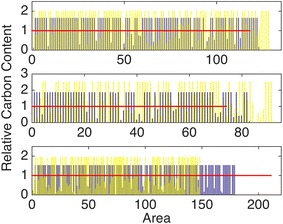
Distribution of carbon content in the global forest areas relative to the initial time after 50 (*blue*) and 100 (*yellow*) years. Each bar represent a forest area of 10500 ha within the boreal (*top*), temporal (*middle*) and tropical (*bottom*) regions. The *red lines* mark the carbon content and number of inital areas in the simulations

The instantaneous status of the forest areas initially, after 50 and 100 years illustrates the development of the forest regions in this simulation (Fig. 3). Each bar represents 100 randomly merged simulation areas of each region, i.e., a forest area of 10500 ha. The height of each bar represents the relative carbon content in these areas normalized to the initial situation (red line). After 50 years, we observe some growth in areas without harvesting, and some regions of significantly less carbon content where harvest has taken place. After 100 years we observe that the carbon content of the untouched forest areas has increased even further while the number of harvested regions has increased as well. Finally, the initial number of forest areas has decreased in the tropical region due to deforestation while forest areas have increased in the temperate and boreal zones, due to afforestation.

**Fig. 4:**
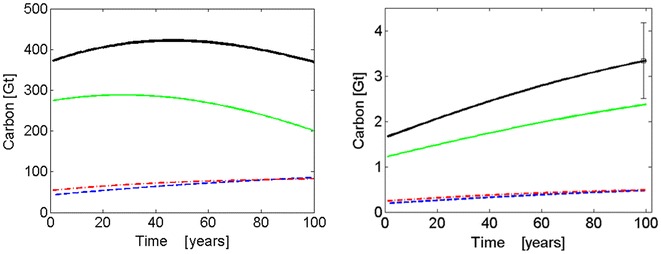
*Left panel* Total forest carbon development in tropical zone (*green full*), temperate zone (*blue dashed*), boreal zone (*red dashed-dot*) and world forest (*black thick full*) *Right panel* Yearly harvested carbon development in tropical zone (*green full*), temperate zone (*blue dashed*), boreal zone (*red dashed-dot*) and world forest (*black thick full*) indicative error bar (*square*) at year 100. Parameters: harvesting rate of 0.45 % in all zones; deforestation rate of 0.3 % in tropical zone; current expansion rates of 0.1 % in the boreal zone and 0.264 % in the temperate zone

The more aggressive harvest strategy in simulation 2 (without additional afforestation areas or actions to reduce tropical deforestation) may not be sustainable (Fig. 4). Here, more than 2 GtC is harvested, with a harvest rate of 0.45 % per year. The forest carbon stock will still, after 100 years, maintain its original state. Note, however, that the forest biomass will decrease towards the end of the period. Thus, the limit for what can be extracted globally every year is about 2 GtC in a 100 year perspective.

**Fig. 5:**
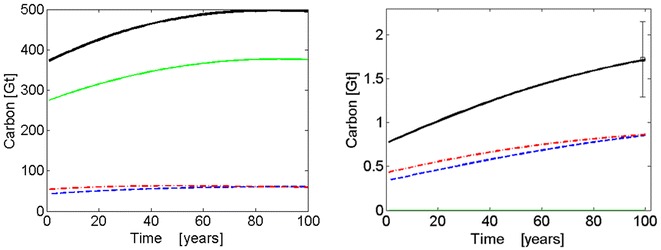
*Left panel* Total forest carbon development in tropical zone (*green full*), temperate zone (*blue dashed*), boreal zone (*red dashed-dot*) and world forest (*black thick full*) *Right panel* Yearly harvested carbon development in tropical zone (*green full*), temperate zone (*blue dashed*), boreal zone (*red dashed-dot*) and world forest (*black thick full*) with an indicative error bar (*square*) at year 100. Parameters: harvesting rate of 0.8 % in boreal and temperate zones; no harvesting in tropical zone; deforestation rate of 0.3 % in tropical zone; current expansion rates of 0.1 % in the boreal zone and 0.264 % in the temperate zone

In the final two simulations we extract carbon only from temperate and boreal zones to protect the tropical forest. After the simulation period, boreal and temperate zones retain almost the same amount of forest carbon as today, in the regime of annual harvest rate of 0.8 % (Fig. 5). In total, forest carbon grows from 372 to 496 GtC and the extracted average amount is about 1.25 GtC per year, with a total of 2.5 GtC sequestrated by both standing stocks and harvested wood every year.

In simulation 4, storage may be raised further if an additional planting program adds 0.05 % of the original area in boreal and temperate zones every year while in tropical zone the deforestation rate is reduced from 0.3 to 0.25 %. The forest area then increases by 80 million ha. Under these assumptions the total harvested wood remains the same while the carbon content of the standing stocks increases up to 526 GtC, or 1.5 GtC per year. The total amount of sequestration is about 2.8 GtC annually.

The uncertainty in the estimates above stems partly from the model itself and partly from external factors. The model error bars are mainly determined by the deviation of the applied growth curves from the real average growth characteristics of global forests. Comparison with available growth data indicates that this uncertainty is in the order of 20 % or less. Additional caveats come with external factors: future technology improving the growth may occur on one side; deterioration of the soil or lack of nutrients or water supplies hindering growth may occur on the other. Regional catastrophic events such as wildfire, insect attacks, wind throw or volcano eruptions and the like may also happen. However, considering the annual global forest area change rate of $-$0.165 % from 1990 to 2010 [25], we believe that external factors in total lead to less than 5 % additional uncertainty. We thus conclude that the final figures above come with a total uncertainty of about 25 % reflected by the uncertainty of the growth model and indicated by black error bars at year 100 in the preceding figures.

## Conclusions

We have developed a dynamic model for carbon storage in forests and harvested wood as an active CCS strategy. With harvest parameters limited by the need to conserve the forests, applications of the model show that in a 100 year perspective the capacity of harvested wood is 1–1.5 GtC per year, and the total sequestration including storage in living trees is on average more than 2–2.5 Gt carbon per year. This is in fair agreement with the previous estimate of Zeng [12], but it also indicates that the harvest estimate is at the upper edge of what is possible without a serious reduction in total forest areas.

Our model also shows that the amount of stored carbon depends critically on the strategies for harvesting and planting. As compared to pure forest conservation with no harvest, the advantage of an active harvest strategy is that it may be applied at a constant rate on a timescale of several hundred years, not being constrained by maturing forests that cannot store more. Also, this proposal does not change the albedo since the small percentage areas of harvesting are rapidly becoming green again after the replanting. Our obtained numbers are a factor of five smaller than Zeng’s [11] estimate of carbon storage potential from collection of dead and mature trees. The latter requires harvesting from the entire world forest areas, while the present plant and harvesting (PH, described in the Methods section) strategy is performed in concentrated regions of less than one percent of the global forest area.

A main advantage of implementing the proposed approach is storage at low costs. For industrial CCS, estimated costs are in the order of 100 USD per tonne of CO$_2$, which are about 150 % of the costs of electricity generation by fossil fuels [5]. Compared to this, harvesting and storing wood will very likely be competitive and the upper estimate is between 25 and 50 USD per tonne of CO$_2$ [13]. In conclusion, we believe that the present results are relevant in a medium-term future scenario of continued and even increased consumption of fossil fuels.

## Methods

In this section we derive the plant-harvest (PH) model used in the simulations and assess its validity in comparison with tree growth data. The idea in our approach is to develop a characteristic average growth curve of wood at a latitude and to discretize the forest area at each latitude. The PH-model associates forest growth potential worldwide with different temperature and latitude characteristics in three zones: tropical, temperate and boreal. Within each zone the wood volume growth follows this characteristic curve after planting. Every year a certain area is harvested and immediately replanted. Additionally, new areas may be opened up for afforestation.

The global forest volume in cubic meters is computed as a function of time,

1 $$\begin{aligned} F_A(t) = F_A^0 + \sum _{i=1,2,3} \int _{t_0}^t \left[ G_i(t')-H_i(t')\right] dt' \end{aligned}$$

Here $F_A^0$ is the initial global forest volume [m$^3$] and $G_i$ and $H_i$ are the volume growth [m$^3$] and harvest [m$^3$ year$^{-1}$ ] in zone *i*.

The growth functions are non-linear and determined mainly by the incoming radiation, the CO$_2$ concentration and the availability of water and nutrients, in addition to the age of the tree stand within each area. Equations describing the growth characteristics of specific trees are in general empirical in their origins, such as the logistic equation or its generalization, the Richards equation [27]. Other applied growth curves are the Gompertz model and the modified Weibull model [28]. For a global approach, it is necessary to represent the forest growth within each area in terms of a characteristic growth function. This may be inappropriate when describing the growth of a single tree, a single stand, or a particular species, but quite accurate in terms of the expected large-scale carbon production stored in wood over time. We start out by deriving this growth model for that purpose.

Let *T* be the age at which planted trees within a large area start to spend all of their energy on maintaining the total mass, so that volume growth after *T* is effectively zero within the area. During the time interval (0, *T*) the wood volume reaches its maximum size, i.e., $V(t) \in (0,V_{max})$. Growth is generated by a total area of leaves being exposed to incoming electromagnetic radiation. Thus, the volume growth can be assumed to be proportional to the exposed area *A*(*t*) set up by the leaves of all trees within the given fixed region,

2 $$\begin{aligned} \frac{dV}{dt} = \epsilon (t) A(t) \end{aligned}$$

Note that both the exposed area *A* and the proportionality factor are time-dependent: *A*(*t*) increases with the volume growth of the trees and is taken in the following to be proportional to the total wood volume, $A(t) = \alpha V(t)$. However, for the photosynthesis to be active, each plant uses energy internally, for instance for transport of water and other molecules up to its leaves. Some of these costs will increase with the size of the tree. At the time when the stand has reached its maximum size the proportionality factor is zero, $\epsilon (T) \simeq 0$. We will here assume a linear dependence from its initial value,

3 $$\begin{aligned} \epsilon (t) = \left( 1 - \frac{V(t)}{V_{max}} \right) \end{aligned}$$

Thus we have obtained a logistic equation for wood volume growth within an entire region

4 $$\begin{aligned} \frac{dV}{dt} = \alpha V(t) \left( 1 - \frac{V(t)}{V_{max}} \right) , \end{aligned}$$

where the two proportionality constants have been merged into a single time independent parameter $\alpha = \epsilon _0 \epsilon _1$. (We here side-remark that by assuming a non-linear efficiency function for $\epsilon (t)$, the Richardson equation can be obtained). The solution of Eq. (4) is,

5 $$\begin{aligned} V(t) = \frac{V_{max}}{1+e^{-\alpha (t-t_p)}} \end{aligned}$$

where $t_p$ is the time at which the volume growth is at its largest. Next is to find a reasonable way to estimate $V_{max}$.

According to the World Energy Assessment 2000, the net energy yield (E$_Y$) for wood is in the range of 30–80 GJ/ha/year [29]. E$_Y$ is what the forest has converted to bioenergy, in terms of wood and what can finally be harvested after a period of time. In this paper we apply 76, 62 and 38 GJ/ha/year for the tropical, temperate and boreal region respectively. Based on existing studies, we take *T* for each zone to be 200, 150 and 140 years [30]. It is reasonable to assume an average dry wood density $\rho _{w}$ of 0.6 tonne/m$^3$. The calorific value of wood $(C_w)$ depends on wood type, but in concordance with this approach we apply a value of $20 \cdot 10^9$ J/tonne for all three zones.

6 $$\begin{aligned} V_{max} \cdot \rho _{w} \cdot C_{w} = E_Y \cdot T \end{aligned}$$

As a consistency check of the present numbers we compare the computed $V_{max}$ from Eq. (6) with the data for power density of tropical plantations and commercial boreal forestry in [31]. In the first case, taking a burn efficiency of 35 %, our computed $V_{max}$ corresponds to an energy density of 0.7 and 0.34 W/m$^2$ in the tropical and boreal regions respectively. The corresponding numbers from Ref. [31] are in the range 0.6–1.1 W/m$^2$ in the tropical regions and around 0.3 W/m$^2$ in northern Europe wood forestry. Thus, $V_{max}$ is pinpointed by a single parameter $E_Y$ and the total growth time *T* taken from Ref. [30]. The other free parameter is the proportionality factor $\alpha$ of Eq. (4). The numerical values of input parameters and growth curve parameters are summarized in Table 1.

**Table 1:** Extracted total growth time *T*, net energy yield $E_Y$, computed growth model parameter $V_{max}$ (m$^3$/ha) and applied growth rate parameter $\alpha$ of each region

Region	*T* (years)	$E_Y$ (GJ/ha/year)	$V_{max}$ (m$^3$/ha)	$\alpha$ (1/year)
Tropical	200	76	1262	0.013
Temperate	150	62	775	0.015
Boreal	140	38	406	0.02

**Fig. 6:**
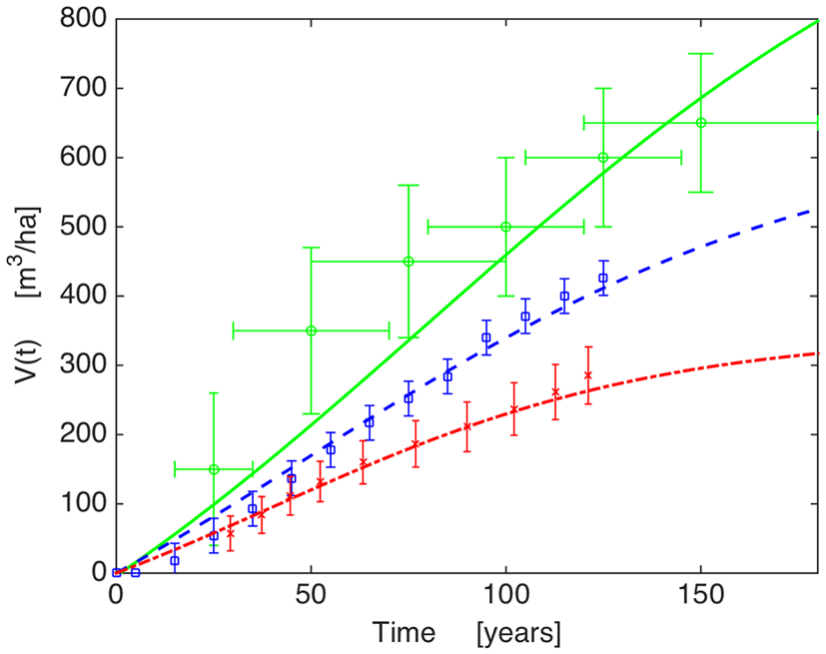
Growth models in comparison to each other and to forest data: *Green* (*full*) curve: Tropical zone growth curve, cf. Table 1. *Green bullets* with error bars are tree height-diameter data from Brazil [34]. *Blue* (*dashed*) curve: Temperate zone growth curve. *Blue boxes* are data from USA [32]. *Red* (*dashed-dot*) curve: Boreal zone growth curve. *Red crosses* are data from Norway [33]

The resulting growth curves of each zone to be applied in the PH model (Fig. 6) may be compared to existing data sets, even if they cannot be expected to reproduce the growth of a single stand or the forest of a localized area very accurately. The data points refer to compiled data from the literature. We start out to discuss the comparison with the most extensive data set we could find covering the temperate zone (blue squares). These come from US forests covering the West and East coast areas [32]. We here select representative temperate forests species from Northeast, Northern lake states, Northern prairie states, Pacific Northwest, Pacific Southwest and Rocky Mountain South respectively. The data consists of time series from year 5 to year 125 measured every 10 years of the forest stand yield (in (m$^3$ /ha) of a certain species or species combination after reforestation. The average growth data of these samples are in good agreement with our temperate growth curve. The error bars, calculated from measurement and sampling uncertainty are below 25 m$^3$ /ha at all times.

Similar extensive forest data could not be found in the two other regions. However, we may compare the regional growth curve with data for single stands. This is a smaller problem in boreal forests than in the other regions, because boreal forests are often dominated by only a few species. However, local environmental conditions will always have impact on growth. As an example, we compare stand growth data for Norwegian spruce with the boreal growth curve from [33]. Typical data points with error bars covering the spread of tree data have been extracted and multiplied again by a timber value of 0.2. In this case the boreal growth curve is in agreement with the data as well. For the tropical region (green), the open circles are average diameter at breast height data extracted from São Paulo State Park of Serra do Mar in Southeastern Brazil, 400 m above sea level [34]. The stored carbon volume per ha is calculated from 1500 stems ha$^{-1}$, an average tree volume of 6 m$^2$, a timber value of 0.2 and a linear relationship between the diameter at breast height (0–75 cm) and the age (0–200 years). Since these are measurements of individual trees, the transformation to wood volume as function of age gives an error bar along both axes. The indicated error bar covers the scattered data quite reasonably [35]. With these values our growth model is seen to fit relatively well with existing data.

In summary, the comparison with average wood growth from the regional USA data indicates an uncertainty in the growth model of less than 20 %. The model performs well with individual stands and tree data samples in the boreal and tropical region as well. However, we cannot deduce an absolute model error unless large data sets over a 100 years period from all regions had been available. The fact that the growth curves agree well with the data actually available supports the assumption of an accuracy of the order 20 % which induces a similar uncertainty in the simulation results.

With the growth functions at hand, the model propagates in time, year by year according to different assumptions of harvest rate, deforestation rate and expansion rate. In the start each discretized forest area has the same fraction of the total known carbon content of each of the three forest zones. This automatically sets the initial age where each area continues to grow from. The expansion rate of each forest zone decides the number of added area units being created every year. Once created, the new area will start accumulating carbon according to our growth function from zero time. Deforestation rates, only valid for tropical zone, determine how many areas will be deleted from the model each year. It is assumed that the carbon stored in these areas will be released immediately since most wood from deforested areas are burned as fuel directly. Harvest rates determine the areas in which the carbon is harvested and stored as wood. After harvesting from one area unit it is assumed that the unit is replanted and continues to grow according to the growth curve from time zero. The total dynamic forest carbon is calculated by summing up the carbon content in all active growing areas of each region each year.
